# Deep Brain Stimulation, Authenticity and Value

**DOI:** 10.1017/S0963180117000147

**Published:** 2017-10

**Authors:** JONATHAN PUGH, HANNAH MASLEN, JULIAN SAVULESCU

**Keywords:** authenticity, deep brain stimulation, anorexia nervosa, autonomy, well-being

## Abstract

Deep brain stimulation has been of considerable interest to bioethicists, in large part because of the effects that the intervention can occasionally have on central features of the recipient’s personality. These effects raise questions regarding the philosophical concept of authenticity. In this article, we expand on our earlier work on the concept of authenticity in the context of deep brain stimulation by developing a diachronic, value-based account of authenticity. Our account draws on both existentialist and essentialist approaches to authenticity, and Laura Waddell Ekstrom’s coherentist approach to personal autonomy. In developing our account, we respond to Sven Nyholm and Elizabeth O’Neill’s synchronic approach to authenticity, and explain how the diachronic approach we defend can have practical utility, contrary to Alexandre Erler and Tony Hope’s criticism of autonomy-based approaches to authenticity. Having drawn a distinction between the authenticity of an individual’s traits and the authenticity of that person’s values, we consider how our conception of authenticity applies to the context of anorexia nervosa in comparison to other prominent accounts of authenticity. We conclude with some reflections on the prudential value of authenticity, and by highlighting how the language of authenticity can be invoked to justify covert forms of paternalism that run contrary to the value of individuality that seems to be at the heart of authenticity.

Deep brain stimulation (DBS) is a highly invasive neurosurgical procedure that has been shown to have profound therapeutic effects in the treatment of movement disorders. In addition to being routinely commissioned for Parkinson’s disease and dystonia in the United Kingdom, DBS is currently being considered as an experimental intervention for a wide range of indications, including certain psychiatric disorders, such as anorexia nervosa and depression.^1^

The majority of patients who undergo DBS for Parkinson’s disease and dystonia experience positive treatment outcomes.^2^ However, even though it is routinely commissioned for these indications, DBS can, in some cases, have unintended adverse side effects.^3^ In particular, a small number of patients have reported feelings of self-alienation following DBS treatment, and some have even seemingly undergone radical changes in their personalities, becoming far more impulsive, and developing tastes and behaviors that they only exhibit under the influence of stimulation.^4^ Although comparatively rare, such cases have been of considerable interest to bioethicists, in large part because of the questions that they raise regarding the philosophical concept of authenticity^5^ (that is, the property of living in accordance with one’s “true self”), as well as questions related to *inter alia*, personal identity,^6^ and moral responsibility.^7^ Conversely however, other patients claim that DBS treatment has enhanced their ability to live authentically, by virtue of removing the disease state that had previously inhibited their ability to live in accordance with their true selves.^8^

The issues related to authenticity are arguably more complicated when we consider the use of DBS in the treatment of psychiatric disorders.^9^ As Alexandre Erler and Tony Hope have observed, some of those with such disorders may view aspects of the self that are regarded as symptoms of the mental disorder as inauthentic; however, other patients may hold precisely the opposite view.^10^ Moreover, the very aim of DBS in this context may be to try to evoke changes to some of the values, beliefs, or affective responses that might be deemed pathological, or to undergird the patient’s disorder. In earlier work, we have tried to address some of the issues pertaining to authenticity in the context of using DBS as an experimental treatment for anorexia nervosa.^11^ In this work, we defended a *diachronic conception of authenticity*, and a corresponding approach to its assessment, according to which patients are encouraged to reflect on changes to their moods and behavior both when “on” and “off” stimulation, to better determine whether the patient embraces them as authentic over time.

This initial discussion has been fruitfully taken up and further advanced in an article by Sven Nyholm and Elizabeth O’Neill in this journal.^12^ Here, we hope to further advance this discussion by exploring the differences between our interpretations of authenticity in the context of using DBS in the treatment of psychiatric disorders. We will begin by briefly introducing the concept of authenticity, before summarizing some areas of seeming theoretical disagreement between our diachronic conception of authenticity, the synchronic approach endorsed by Nyholm and O’Neill, and the broadly essentialist “true self” view advocated by Erler and Hope. We will then consider the practical implications of these disagreements for understanding of the issues pertaining to authenticity in context of using DBS to treat anorexia nervosa.

## Introducing Authenticity

As an initial starting point, we can say that to be authentic is to live in accordance with one’s “true self.” If such language of a “true self” is to be of any practical significance, then it seems that one must also accept that there can be elements of a person’s self more generally that are *not* part of the “true” self, but instead merely peripheral. To live inauthentically is to fail to live in accordance with the true elements, even if one can be understood as living in accordance with these peripheral elements. The key question for a theory of authenticity is how we should identify those features of the self that are “true,” and those that are peripheral.^13,14^

As Nyholm and O’Neill also recognize, social psychology can give us a number of clues about how we *do* in fact seem to go about identifying these features. In a recent review, George Newman et al. point out that when an individual makes an assessment either about his or her own “true self” or another’s, that person tends to emphasize features that have positive valence, particularly if those features are moral features.^15^ Further, and interestingly for our purposes, research in this area also suggests that these sorts of positive features of the self tend to be understood in an essentialist fashion; that is, they are understood to constitute a “discrete, biologically based, immutable, informative, consistent” characteristic that is “deeply inherent within the person.”^16^

We will refer to this as the essentialist conception of authenticity. According to this sort of view, to live authentically is to live in accordance with this deep essence; the path to authenticity on this account is one of self-discovery of this (usually positive) essence.

However, the mere fact that social psychologists have shown that people tend to make judgements about authenticity in accordance with this model clearly does not entail that this is the correct approach to the question of authenticity. First, it seems plausible that the true self could have negative valence, even if people do not assess their own true self (or the self of another) in this way. More significantly, however, some critics of this approach to authenticity have claimed that the idea that we have a hidden essential self that is waiting to be discovered is deeply problematic, and most likely a fiction.^17^ Drawing on themes from existentialist philosophy, advocates of authenticity who dispute the notion of an essential self have claimed that authenticity should be construed as a form of self-creation. To live authentically, in strong versions of this view, is to *choose* the person that one wishes to become, unburdened by the dictates of a fixed essence. Through this approach, we can identify authentic elements of the individual’s self by identifying those elements that the individual reflectively endorses.^18^

The essentialist and existentialist conceptions have sometimes been understood as representing two poles on a continuum of theories of authenticity,^19^ and even as rival conceptions. For example, in their discussion, Erler and Hope argue that (1) existentialist conceptions of authenticity lack practical utility for those who wish to draw on the notion of authenticity to help guide their choices and commitments, and (2) that those with mental disorders often draw on an essentialist conception.^20^ For Hope and Erler, the purported lack of practical utility associated with the existentialist conception derives from the conceptual difficulty of how individuals may plausibly be said to authentically choose their own characteristics in the way that the existentialist approach seems to demand. We will elaborate on this criticism of the existentialist conception subsequently.

However, as we mentioned, critics similarly raise concerns about the essentialist conception of authenticity, in particular its seeming reliance on a hidden essential self. In view of the fact that each conception of authenticity has both apparent flaws and strengths, we may well feel attracted to both understandings.^21^ Rather than seeking to explain why one sort of conception is more convincing, a more plausible strategy may be to try and seek some common ground between the two. This strategy becomes more plausible once we concede that one need not be committed to strong forms of essentialism or existentialism of the sort that we have caricatured here. Neil Levy captures this point as follows:

We can emphasize self-discovery without holding the empirically implausible notion that the self has a fixed essence; we can point to the fact that people *do* have dispositions and talents and personalities, which fit them better for some activities than for others . . . without committing ourselves to the claim that people are immutable, and even without denying that genuinely profound change is possible. We can emphasize self-creation without denying that change is difficult and always only partial. The ethics of self-creation and of self-discovery are better seen as outlooks on human life; conceptions of how we best live.^22^

We believe that Levy captures an important insight with this framework, which we will henceforth refer to as the dual-basis framework. We return to this framework of authenticity later.

At this point, however, it should be noted that Nyholm and O’Neill do not invoke the terminology of either self-creation or self-discovery in their discussion of authenticity in the context of DBS. Instead, they identify what they take to be six core features of the true self:

1) The true self permeates human thinking and so will affect how stakeholders interpret the results of DBS.
2) The true self is a synchronic notion that permits us to describe effects of DBS on the self that the diachronic concept of personal identity does not.
3) The extent to which the true self is expressed can be a matter of degree.
4) The degree to which persons feel their true self is expressed can be influenced by their modes of functioning, which can be affected by DBS.
5) In some cases, radical transformation can make the true self more fully expressed.
6) Which features are considered characteristic of a person’s true self depends, in an important sense, on which features he or she values.^23^

We agree with much of Nyholm’s and O’Neill’s assessment; however, we will raise some queries about the second and sixth features that they identify. At this point, however, we may observe that the other four features are clearly compatible with the dual-basis framework that we have just sketched. We take (1) to capture the idea that authenticity is often treated as a normative ideal, as something that we have reason (whether prudential, moral or autonomy based) to achieve; the same can also be said of both essentialist and existentialist elements of the dual-basis framework. Similarly, in accordance with (3) and (4), authenticity in either essentialist or existentialist terms may plausibly be said to admit of degree, and this can plausibly be affected by our modes of functioning, and thus by DBS.

Prima facie, feature (5) might seem problematic for the essentialist element of the dual-basis view. If living authentically is to live in accordance with the dispositions, talents, and personalities that one has (even if we do not make the strong claim that these features must be parts of an immutable essential self), how can radical change be compatible with authenticity? This sort of thought has motivated much criticism of the use of various technologies to enhance human mood and cognition.^24^

However, the prospect of radical change need only threaten authenticity in this conception if we assume that a tenet of this conception is that one must *accept* the extant features of the self that one has thus far discovered. However, as Levy points out, this not a tenet of the essentialist view as it has historically been defended; self-discovery might tell us that we need to undergo radical change in order to live in accordance with our essence.^25^ For example, it is quite possible to have an essentialist understanding of the radical transformation of Ebenezer Scrooge in *A Christmas Carol;* according to such a reading*,* the purpose of Scrooge’s hauntings were to help him to discover that his miserly personality did not reflect who he was at a fundamental level.

The potential points of disagreement between our understanding of authenticity and that which is endorsed by Nyholm and O’Neill pertain to their features (2) and (6). Although Nyholm and O’Neill do not invoke the terminology of essentialism or existentialism, we believe that these features of their understanding of authenticity seem to invite an existentialist interpretation of their view as we shall go on to explain and critique in the next section.

## Synchronicity, Diachronicity, and Value

The central feature of Nyholm and O’Neill’s account of authenticity is synchronicity. In this context, synchronicity implies that the authenticity of a trait or desire is not contingent on the history of the agent’s traits or desires: authenticity can be assessed in an isolated time-slice. In understanding authenticity as a synchronic notion (as feature 2 stipulates), Nyholm and O’Neill draw a distinction between authenticity and narrative identity (as well as numerical identity). In contrast to numerical identity, narrative identity concerns the qualitative sense of identity that captures the continuity of a person’s character over time, a character grounded by an autobiographical self-narrative that incorporates the agent’s past traits, actions, and experiences.^26^ As authenticity is synchronic in Nyholm and O’Neill’s account, whether or not a person is authentic does not depend on whether that person exhibits the sort of continuity over time that narrative identity implies.

In the context of DBS, Nyholm and O’Neill are therefore concerned that by focusing only on narrative identity, bioethicists might overlook the question of whether DBS has an important impact on the self *here and now*, independently of how the person relates to him- or herself in the past. They claim: “if a patient has experienced severe OCD over a long period of time, it might be more in keeping with her past narrative if she were to continue having obsessions and compulsions. However, one might think instead that her real self would be better served if she could rid herself of that dominant narrative.”

Claiming that authenticity is synchronic in this way has important implications for the question of how we should ascertain whether some feature of the self (e.g., a particular desire) is authentic, which is a key question for any theory of authenticity. More specifically, understanding authenticity as a synchronic notion seems to require abandoning the essentialist claim that to ascertain whether some feature of the self is authentic, we should appeal to other enduring, extant elements of the self. If authenticity is purely synchronic, why should these *enduring* elements have implications for authenticity in the here and now?

Feature (6) offers some clues as to how Nyholm and O’Neill believe we should ascertain an agent’s authentic desires. On this approach, an individual’s true self may plausibly be construed as being constituted, and indeed grounded, by the agent’s *values*. However, in their discussion of this feature, they do not elaborate on their understanding of values in this context; rather, they focus on how third-party assessments of authenticity will be informed by the third party’s values.

Although it is no doubt true that third-party assessments of authenticity will be informed by the third party’s values, we are more interested in the role that the individual’s values play in that person’s own sense of authenticity. However, without further elaboration on what it means for a person to value something, Nyholm and O’Neill’s appeal to the individual’s values seems to leave their understanding of authenticity open to the critique that Erler and Hope aim at existentialist accounts, briefly sketched previously. To see why, it is illuminating to first consider the main targets of Erler and Hope’s criticism. They aim their criticism at Harry Frankfurt’s wholeheartedness account (according to which an element of the self is authentic if endorsed wholeheartedly) and David DeGrazia’s autonomy-based account. According to this latter account, a self-creation project is authentic if it is both autonomous and honest. In turn, a self-creation project is autonomous if (1) the agent chooses it because that person prefers this project, (2) that person has this preference because he or she (at least dispositionally) identifies with and prefers to have it, and (3) this identification has not resulted primarily from influences that that person would, on careful reflection, consider alienating.^27^

Erler and Hope think that this sort of conception is problematic because it lacks practical value for those who wish to draw on the notion of authenticity to help guide their choices and commitments. They write: “When a person is struggling with the question of what are her authentic desires (or other relevant psychological aspects of the self) it is not particularly helpful to be told that whatever she decides, as long as she commits herself *wholeheartedly* or has *reflected carefully*, will be authentic. The question of authenticity, from this perspective, *precedes* and informs which desires the person wishes to endorse or which decisions she makes.”^28^

We take it that the point Erler and Hope are making here is that existentialist conceptions of authenticity such as Frankfurt and DeGrazia’s arguably put the cart before the horse. Such theories claim that to ascertain whether some element of the self is authentic, we broadly need to consider whether the agent would identify with it after reflection; however, if such reflection is to be a guide to authenticity, we surely need to know that the sort of reflection being conducted is *itself* authentic.

We will not be concerned here with the exegetical question of whether this is a fair criticism of DeGrazia’s conception of authenticity. We believe that it is possible to develop a dual-basis view of diachronic authenticity that builds on the idea of reflective endorsement but that avoids Erler and Hope’s criticism. At this point however, we may note that Erler and Hope’s criticism seems problematic for existentialist authenticity conceived as a purely synchronic notion in the way that Nyholm and O’Neill outline. The reason for this is that if authenticity is a purely synchronic notion, then it is not clear what basis there could be for grounding the authenticity of elements of the agent’s self, including that agent’s present values, other than the values that the agent exhibits *here and now*; however, this is the very element of the self whose authenticity is under question.

## A Dual-Basis View of Diachronic Authenticity

To avoid Erler and Hope’s critique of existentialist approaches to authenticity, we believe that we need to appeal to diachronic values, and in doing so, incorporate some broadly essentialist elements to our account of authenticity. In light of Levy’s comments, however, we take this to be a strength rather than a weakness of our theory. Using the approach that we endorse, we may say that a person values *x* when that person believes that *x* is good, in the sense that that person understands him- or herself to have broadly prudential or autonomy-based reasons to pursue *x*; our values are thus responses to our beliefs about what is good *for us or others*. This is a rationalist approach to value.^29^ Although we can revise our beliefs about what is good for us, it would be indicative of irrationality (or reasons-irresponsiveness) if these beliefs were unduly capricious.

We claim that the true self is best construed as being constituted by the cohering elements of the individual’s nexus of values and that individual’s rational beliefs. Here, we broadly follow a view of authenticity that is implicit within Laura Waddell Ekstrom’s coherence account of personal autonomy. Whereas Ekstrom develops a nuanced account of what it is for elements of the self to cohere, for our purposes here, a rough understanding will be sufficient. Roughly, we may say that these elements of the self cohere if they are mutually compatible. In the case of mutually incompatible elements of the self, such as, say, a desire to *x* and a desire to *y*, individual agents must decide whether it is more valuable for them to realize their desire to *x* or to realize their desire to *y*, given their other coherent values and rational beliefs. If they deem it more valuable for them to realize *x* than *y*, then their preference to realize this desire may be incorporated as a cohering element of their true self.^30^

In developing this account, Ekstrom claims that cohering rationally endorsed elements of the self are good candidates for constituting the true self for three reasons. First, they are particularly long-lasting because they are well-supported with reasons. This is important because, as Ekstrom recognizes in her discussion: “a variety of beliefs and desires . . . come and go in us in a rather fleeting manner. But we expect our character to be more continuous than this – if not constant, then at least not in a state of perpetual fluctuation.”^31^ Second, cohering elements will be fully defensible against external challenge by virtue of their support from the coherent nexus in which they reside. Third, they will also be elements that the agent feels comfortable owning, by virtue of that same fact.^32^

Consider first the implications of this coherence approach to the relationship between narrative identity and authenticity. First, on the rationalist understanding that we have sketched, persons can clearly *disvalue* significant elements of their personal history. Accordingly, with respect to Nyholm and O’Neill’s OCD example, the mere fact that the person’s history has included experiencing the symptoms of OCD, does not tell us anything about the implications that treatment may have for authenticity. In order to ascertain this, we would need to know how the patient values his or her experience of these symptoms. For example, some successful academics might plausibly value their obsessiveness over details of their work; conversely, compulsive hand-washers may want desperately to be rid of their anxiety and compulsive behavior.

To this point, it might be claimed that the coherence approach seems to be an account of authenticity that contrasts the concept with the notion of narrative identity, in so far as we claim that one can disvalue significant elements of one’s personal history. However, this understanding of authenticity departs from Nyholm and O’Neill’s synchronic understanding, in so far as an agent’s values are most plausibly understood in a diachronic sense. We believe that this also helps to explain how a coherence approach to authenticity can avoid Erler and Hope’s criticism regarding practical utility, as we will now explain.

## The Practical Utility of a Diachronic Conception of Authenticity: Enduring Values and Intelligible Change

To begin, it is important to note that the expectation that elements of the true self will be continuous and long-lasting is quite consistent with the possibility of one retaining authenticity despite undergoing a radical change in character (as Nyholm and O’Neill’s feature 5 suggests). Such change can be authentic if it is intelligible to the agent in the light of that agent’s preexisting values and commitments. To illustrate, consider again the example of Scrooge from *A Christmas Carol*. Previously, we explained that it is possible to give an essentialist reading of this example, according to which Scrooge may be understood to be authentic following his radical change, because the hauntings helped him to discover that his miserly personality did not reflect his essence. According to this reading, Scrooge’s change is intelligible to him by virtue of the preexisting deep value (of non-miserliness) that actually constituted his essence, or part of it, and which he comes to accept and recognize as his own.^33^

However, we can also make sense of the importance of preexisting values without committing ourselves to this overtly essentialist interpretation, whereby Scrooge was really *never* a miser. According to a reading that is more in keeping with the existentialist approach, part of the reason that we might believe that Scrooge was living authentically after his radical change is that the ghosts who haunted him *persuaded* him to change his miserly ways by *appealing to other values that he held*, to show him that he had reasons to change his hitherto positive evaluation of “being miserly.” The ghosts showed him that he would die alone and despised if he continued his miserly ways. This strategy would only have been successful if Scrooge, as he appears to do, *already* placed disvalue on a life in which this occurred.

Importantly, according to this interpretation, the change that Scrooge underwent cannot be completely wholesale if it is to be authentic. For Scrooge to believe that he has reasons to change his ways, there must be something in his conception of the good prior to his haunting through which he can understand *why* he has a reason to change; if not, it is not clear how the change could be intelligible to Scrooge. Using this approach, although we can undergo radical authentic change, we can only do so in a manner akin to rebuilding Neurath’s raft; that is, we can only intelligibly and justifiably change constituent parts of our true selves by appealing to other values that we hold. Although we may come to change many or even all of our values over time, such changes are only authentic if our decision to do so is made intelligible by some other reason implying value that we maintain over the course of that change.^34^

It is important to remember that our character contains many elements, some often in conflict with others. Few people are purely virtuous or purely vicious; we are all conflicted, a mix of “light and dark.” Using the coherence approach, the true self is best understood as the set of cohering elements of the self that we understand ourselves to have most reason to preserve. Our choices about which elements of our characters to preserve as central elements of our selves amount to decisions to bring out certain aspects of our character, while downplaying others. In choosing his response to his haunting, Scrooge chose to emphasize the “light,” socially acceptable elements of his character system, and to downplay the “dark,” in rejecting his miserliness.

The truth of essentialism is that we may have certain elements of our character that are more or less fixed. The truth of existentialism is that we may be able to choose which of these more or less fixed elements to bring to the fore, and which to downplay in developing our selves. One of the problems we face when thinking about authenticity in the context of mental disorder is that some “pathological” elements of the self seem to lack value, and do not seem worth preserving. Moreover, with some mental illness, there may be no stable coherent sense of self, and treatment may involve attempts to bring about a stable coherent self.

We will consider the application of our approach to authenticity to mental disorder in greater detail subsequently. At this point, however, the discussion of the Scrooge example helps to explain why the coherence view is not susceptible to the criticism that Erler and Hope raise against DeGrazia’s existentialist conception of authenticity. The coherence approach can give practical guidance to those who wish to draw on the notion of authenticity to help guide their choices and commitments. It is true, with this approach, that simply establishing that the agent endorses some desire in accordance with a rational evaluation is not alone sufficient for establishing authenticity; we can still ask whether the rational evaluation itself is incorporated into the agent’s true self. This is the point that Erler and Hope’s critique of Frankfurt and DeGrazia’s account raises. However, the coherence approach can offer an answer to this question by investigating whether the rational evaluation is incorporated into a coherent character system, whose lineage can be traced back over a diachronic process of intelligible rational change.

The crux of Erler and Hope’s criticism of existentialist accounts seems to be that if the language of authenticity is to be of practical value, there needs to be some sort of foundational essential self whose characteristics can plausibly undergird our judgements of authenticity. This has parallels with the approach that epistemic foundationalists adopt in understanding the justification of knowledge; epistemic foundationalists claim that certain beliefs are basic, and that our other beliefs are epistemically dependent on these basic beliefs. Whereas epistemic foundationalists face difficulties in accounting for the items of basic knowledge, adopting a foundationalist approach to the self faces the analogous problem of stipulating the existence of a foundational, or basic, essential self. The coherence approach that we advocate also has a parallel in epistemology.^35^ Epistemic coherentists do not claim that the justification of knowledge requires basic items of knowledge, but rather that our beliefs constitute knowledge in so far as they belong to a coherent system of mutual justification. Just as epistemic coherentists do not need to stipulate basic items of knowledge, those who adopt a coherence approach to authenticity do not need to stipulate the existence of an essential self.

That said, although the coherence approach is existentialist in spirit, it also incorporates significant elements of the essentialist approach. We have already seen that Ekstrom stresses the long-lasting nature of elements of the cohering self, and the importance of this feature. A second point that Ekstrom does not acknowledge but that is apposite here is that we do not develop our values in a vacuum; our beliefs about what we have reasons to pursue are likely to be informed by fixed elements of our lived experience, including our awareness of our past experiences, and the set of traits and dispositions that we have, in part in virtue of our biology. The extent of our self-creation is thus limited: authentic change on this account is difficult and always only partial in the manner that Levy raises in his discussion of what we call the dual-basis framework. Our values and essential elements of our characters may thus be understood in a symbiotic fashion; it is through the lens of our evaluations, themselves developed in the light of our personal history and our stable, long-lasting characteristics and traits, that we are able to understand which of our features we want to be incorporated into our understanding of who we really are.

## Authenticity of Values versus Authenticity of Traits

So far, our discussion has included a range of objects of authenticity: (1) the agent him or herself, (2) the agent’s traits and characteristics, and (4) the agent’s rationally endorsed desires and values. To a certain extent, these are interrelated: we often (although not always) exhibit traits and behaviors that reveal our values: if an agent is consistently conscientious at work, this may be explained by the value that the person places on the ends of his or her toil, or on working hard *per se*. Conversely, our biological and psychological makeup is likely to have some influence on our values: in general, an agent who is naturally gifted with an athletic physique may come to value athletic activity and excellence more than an agent who substantially lacks athletic prowess. In relation to authenticity, we noted in the previous section that we do not develop our values in a vacuum, and that our values and essential elements of our characters may be understood in a symbiotic fashion.

However, the relationship between our traits and our values is clearly not a determinate relationship. We can disvalue aspects of our character and behavior, and our values can generate and sustain rationally endorsed desires that motivate behavior that resists the influences of more basic drives and urges. Therefore, despite the inevitable interaction between traits and values, we will argue in this section that there is still an important distinction to draw between the authenticity of our more essential traits on the one hand, and the authenticity of our values on the other, with significant implications for how troubled we should be by the effects of a DBS intervention (or the effects of a psychiatric condition). The dual-basis framework, which acknowledges the essential nature of many of our traits, yet allows for authentic rejection or modification of these traits, supports this distinction.

We will argue that we should be most concerned about DBS interventions that affect the authenticity of an agent’s values, especially where these values inform treatment decisions. Interventions that affect the authenticity of an agent’s traits, on the other hand, are only problematic in so far as the agent, all things considered, (authentically) disvalues this influence. We now illustrate this distinction and its implications with two examples.

### Case One: Inauthentic Traits

A 70-year-old man with advanced Parkinson’s disease underwent DBS of the subthalamic nucleus (STN). The patient developed hypersexuality as a side effect, insisting on sexual gratification from his partner. Once satisfied, the patient returns “back to his normal self,” and confronts the realization that he could not control his (unwanted) urges.^36^

In this case, it appears that the DBS treatment generated inauthentic urges and related behavior (hypersexuality), but did not affect the patient’s values relating to those urges and behaviors. We can assume that, prior to the intervention, the patient’s values did not generate rational endorsement of hypersexual behavior, and the case report suggests that the patient continued to disvalue such behavior, which he now found himself engaging in following stimulation. This motivating urge was incongruous with the agent’s own nexus of values and beliefs.

### Case Two: Inauthentic Values

Apathy has been observed as a postoperative symptom of STN stimulation surgery. Apathy can be measured using the Frontal Systems Behavior Scale, which measures apathy using items such as “Has lost interest in things that used to be fun or important to him/her,” “Shows little emotion, is unconcerned and unresponsive,” and “Has difficulty starting an activity, lacks initiative, motivation.”^37^

A DBS treatment that resulted in a significant increase in a patient’s apathy might have a direct impact on the patient’s values; apathy can be characterized as a failure to be moved to express or act on one’s values. We suggest that a treatment that impacts on the patient’s values in this way is more problematic than a treatment that renders only (a number of) the patient’s traits inauthentic. In case one, the patient’s inauthentic hypersexual urges were clearly undesirable, not least for the patient himself. However, in this case, the patient was in a position to decide whether the benefits of the intervention (reduced PD symptoms) outweighed the cost of the inauthentic urges and associated behaviors. Therefore, even if the DBS treatment has an effect on the patient’s authenticity (as it pertains to that patient’s traits), this aspect of inauthenticity would only rule out continuing with the DBS treatment if, *from the patient’s assessment of his or her best interests,* the harms of the treatment outweighed the benefits. As we will explore, this example may be an instance in which authenticity (at least of traits) is less relevant than the question of what, overall, leads to the better life for the patient. However, although the incidence of inauthentic traits does not necessarily provide a decisive reason against continuing a DBS treatment, we do not suggest that inauthentic traits are *irrelevant* to treatment decisions. Further, inauthentic values have acute relevance, as we now argue.

In case two, inauthentic values (or lack of authentic values) flowing from a significant increase in apathy would, we argue, provide a much stronger reason against continuing with the treatment, especially where the inauthentic values inform the treatment preferences of the patient. For example, if, as a result of increased apathy, the patient expressed a preference to continue with the treatment, because that patient did not care about the broader effects (including the increased apathy), then this treatment preference should be treated as much less instructive. This will especially be the case if the treatment preference is in tension with preferences expressed “off” stimulation. Consider also a case in which a patient develops hypersexuality under stimulation but does not regard this behavior as abnormal, and perhaps even endorses this change. In these cases, the normative significance of the inauthenticity of the patient’s values differs from the significance of the unpleasantness of exhibiting inauthentic traits. The patient, with the patient’s physician and family members, can evaluate the inauthentic traits resulting from DBS, whereas inauthentic values resulting from DBS affect the very grounds of the patient’s treatment decisions.

In the next section, we will turn to examining how the diachronic approach to assessing authenticity bears on the case of anorexia nervosa.

## Authenticity and Anorexia Nervosa

From the outset, it seems that the coherentist approach faces a significant difficulty. In many cases of psychiatric disorders, the condition itself can plausibly be understood to distort the patient’s values with implications for that patient’s corresponding authenticity. Moreover, individuals with such disorders very rarely have a coherent sense of self, and are instead subject to feelings of extreme self-conflict. Erler and Hope stress this point, and suggest that these individuals draw upon the idea of authenticity to help find a way to resolve the conflict and give direction to self-development. In turn, they suggest that there are five alternative positions that individuals with mental disorders such as anorexia nervosa seem to endorse with respect to authenticity, as follows:

1) The authentic self is the well self and aspects of the self that are part of the mental disorder are inauthentic.
2) Psychological characteristics that result from taking medication are not authentic.
3) Mental disorder is part of a unified self; they see their disorder as an authentic part of who they are.
4) There are two selves, each equally authentic.
5) There is no issue of authenticity: the only consideration is what leads to the better (or best) life, and questions of authenticity are irrelevant to that question.

However, we will argue that the prevalence of inner conflict in persons with mental disorders such as anorexia nervosa, and these different ways in which such individuals draw on the concept of authenticity, do not speak decisively against adopting a coherence approach to authenticity in this context. Indeed, as we suggested in our discussion, we are all conflicted to some degree.

The first thing to note is that, as a procedural account of authenticity, the coherence approach is compatible with either (1) or (3) being true of a *particular* individual. Some essentialist views of authenticity only advocate something like (1) as being true for *all* individuals with anorexia nervosa; for example, Jacinta Tan et al. have argued that the anorexic patient’s extreme positive evaluation of low weight is not authentic because it is a “pathological value.”’^38^ With this sort of approach, the authenticity of certain elements of the self can be determined by their substantive content; a strong desire to maintain an extremely low weight is necessarily inauthentic because that desire is itself part of the pathology of anorexia nervosa. One benefit of this approach is that it provides an approach to authenticity that offers clear practical guidance to those with mental disorder. However, the problem with this approach is, as Erler and Hope observe, that many persons with mental disorder claim something like (3); they believe that their “pathological values” are part of their authentic self.

The substantive approach resolves this conflict in favor of a view of authenticity that reflects (1), stipulating that pathological values cannot be held authentically. This strategy gives authority to the healthcare provider over the patient herself with regard to the question of the authenticity of the patient’s internal states. We have argued elsewhere that this strategy is problematic;^39^ however, it is important to be clear that the coherence approach does not similarly resolve the conflict in favor of a view of authenticity that instead reflects (3) simply by fiat. To simply say that what the patient herself “feels” or “believes” at a nonreflective level has unquestionable authority with regard to the authenticity of elements of her self would be problematic, given the high degree of inner conflict and vacillation that such patients experience with regard to this very issue. Rather, in cases in which these “pathological” values may plausibly be understood to have been incorporated into the agent’s authentic self-understanding, this must be grounded by the coherence of those values with other long-standing cohering elements of the agent’s character system, elements that are rationally intelligible to that agent.

Establishing that this is the case requires going deeper than simply asking what the patient herself believes or feels at a given moment. It may require investigating the reasons why she holds the desires she does, and how the values that undergird those desires relate to other values and beliefs that she holds. This strategy may help to elucidate whether these desires are grounded by the patient’s own rational endorsements, and whether they have any basis in reality. Moreover, it may serve to tease out potential inconsistency and conflict. However, with this approach, particularly if such conflict does not exist, it is quite possible for an agent to authentically hold the values that are characteristic of anorexia nervosa as part of her self-conception, particularly in the case of chronic sufferers who may have shaped and developed a coherent character system over many years to accommodate this “pathological” desire.

Position (4) perhaps raises a deeper problem for the coherence approach; namely, that two cohering selves with radically different evaluative perspectives might plausibly reside in the same agent. Such an agent may thus lack stable values. Here, with the coherence approach, authenticity must partly be a matter of self-discovery, in so far as the agent must identify the distinct aspects of her coherent selves; however, it must also be a matter of self-creation, in so far as the agent must decide which of those selves to prioritize as her most authentic self. This is where the crux of the problem lies for the coherence approach in such cases: on what basis can the individual make this decision? The very values that she might appeal to in order to justify her decision are bound up in the very character systems that she may be choosing between.

The coherence approach cannot offer an easy answer in such cases of inner conflict; however, this is perhaps a fitting response to such hard cases. At least the coherence approach may allow third parties to offer some practical guidance about how the individual might go about making this decision, perhaps by drawing her attention to the strength of certain reasons, and the goods at stake in her decision. Furthermore, it is notable that such cases also raise significant issues for the essentialist perspective. Although the essentialist might claim that there is a right and wrong answer to the question “which of the two selves is the authentic one?” the essentialist still faces the epistemological question of how we should arrive at the correct answer to this question.

Nyholm and O’Neill suggest that in this sort of case, we should assume that the value set that is widely endorsed by others is the authentic one. We will raise our doubts about this response at the end of this section. Prior to doing so, we will first briefly consider the other positions identified by Erler and Hope.

The coherence approach also provides a basis for position (2). Although authenticity is compatible with radical change with this approach, for such change to be authentic it must be rationally intelligible to the agent, as we explored in the previous section. Interventions that serve to directly induce psychological changes, such as psychoactive drugs or DBS, may in some cases result in feelings of alienation because they cause the patient to undergo changes that are unintelligible to that patient, in the light of the patient’s other values and beliefs. Depressed patients who takes Prozac may feel alienated from their elevated mood if the drug serves only to increase their positive affect without engaging with other elements of their character system that may play a role in their condition (such as apathy and feelings of worthlessness). This stands in contrast to indirect interventions that aim to evince changes in the patient’s mood by rationally engaging with the patient, for example, in talk therapy.^40^ Changes brought about via such interventions will more likely be intelligible to patients, in so far as they are brought about by changes that the patients themselves decided to make to their modes of thinking.

Nonetheless, other patients on psychotherapeutics such as Prozac claim that it enables them to find their true self, presumably by creating intelligible changes, possibly rooted in primitive existing aspects of their own psychology. Therefore, this is not to say that all directly induced psychological changes must be experienced as alienating with this approach. In cases in which the patient has consented to a direct intervention, the psychological change induced may be understood as intelligible to the patient in the light of the values that moved them to consent to treatment. For example, suppose a patient consents to undergo DBS for anorexia nervosa. If stimulation is successful in reducing her desire to maintain a low weight, the patient may understand this change in her evaluative stance as intelligible to her in light of her prior desire to change, even if the precise (direct) mechanism by which the change occurred is not intelligible to her.^41^

The final position acknowledged by Erler and Hope, according to which “there is no issue of authenticity” is a position that is best understood as one regarding the value of authenticity and the role it should play in treatment decisions, rather than a position about the nature of authenticity per se. As such, the position is compatible with the coherence approach that we have outlined here, although it is perhaps in tension with the first feature of Nyholm and O’Neill’s claim that authenticity is treated as a normative ideal. To conclude we will offer some further reflections on the role that authenticity plays in well-being, and the reasons that those with mental disorder or their care team give for holding the view that authenticity is irrelevant to treatment decisions, or at least less relevant than the patient’s welfare.

## Authenticity and Well-Being

Authenticity might plausibly be understood to have instrumental prudential value for well-being.^42^ This is perhaps most obvious in hedonistic accounts of well-being, according to which what would be best for someone is what would make that person’s life happiest. Authenticity is plausibly instrumental to well-being because it involves the experience of a particular kind of positive mental state (that of feeling authentic), or at least the absence of a negative mental state (that of alienation).

However, although authenticity is often explicated in phenomenological terms, our positive evaluation of authenticity is not, it seems, *wholly* explicable in such terms. The reason for this is simply that authenticity need not be experienced as a pleasurable mental state, a point that Felicitas Kraemer also recognizes in her discussion of authenticity and DBS;^43^ conversely, alienation need not be experienced as a negative mental state. In such cases, if we still value the experience of authenticity (and disvalue alienation), this cannot be explicated in purely hedonic terms.

One way to capture the value of authenticity in such cases is to understand authenticity to be prudentially valuable as an end in itself, or to be a constitutive element of objective well-being. Using such an approach, our prudential reason to live authentically is not just that it will, on balance, lead to more pleasurable mental states (although it may); rather, we have a reason to live authentically for authenticity’s own sake. This view garners support from John Stuart Mill’s famous defense of individuality as one of the elements of well-being in Chapter 3 of *On Liberty*. Here, Mill writes that the man who cultivates his individuality becomes more valuable to himself, and achieves a “greater fullness of life about his own existence.” Whereas there is some debate about how best to construe Mill’s conception of individuality, some passages hint toward a reading that suggests that individuality for Mill comes close to the conception of authenticity that we have outlined here. Throughout the chapter, Mill defends the importance of developing one’s own character and living in accordance with it, stating that living in accordance with one’s own character is to be understood as living in accordance with the desires and impulses that express one’s “own nature as it has been developed and modified by his own culture.”^44^

Evidence from modern-day social psychology suggests that people tend to echo this Millian view in their understanding of the value of authenticity.^45^ This literature suggests that people value authenticity because it plays a central role in “giving meaning to their lives.” Such an understanding of the value of authenticity fits neatly with Mill’s observation that: “If a person possesses any tolerable amount of common sense and experience, his own mode of laying out his existence is the best not because it is the best, but because it is his own mode.”^46^

With this in mind, consider now the justification that individuals offer in favor of position (5): Jams Hughes, one of the advocates of this position in the context of using medication for attention-deficit/hyperactivity disorder (ADHD) discussed by Erler and Hope, denies the importance of authenticity and claims instead that “The real question for me is whether the drug makes the taker happier and more able to accomplish life goals.”^47^ Hughes implicitly seems to endorse a theory of well-being that incorporates hedonistic elements (in so far as feeling happy is central to what matters to him) as well as elements of a desire-fulfilment approach to well-being (in so far as it is important that they are able to accomplish their life goals). In light of the previous discussion of the value of authenticity, this approach to well-being might seem impoverished if it is understood to eschew all reference to authenticity; for example, we might wonder to what extent accomplishing a goal increases well-being if it is not an expression of one’s own character. However, as distinguished, one can express one’s character by choosing between modes of living and experiences that are open to oneself (including those made open by biomedical intervention), even where some of these are less aligned to one’s more biologically immediate dispositions.

Those who endorse position (5) might plausibly raise the complaint that even supporters of authenticity should concede that it is not the *only* prudential value. In cases of mental disorder, it may also be a prudential value that is incompatible with other plausible constituents of well-being, including, for example, the experience of positively valenced mental states. Considering the precise role of authenticity in well-being would take us far beyond the scope of this article. However, we believe that this brief reflection on this matter raises a concern about Nyholm and O’Neill’s preferred strategy when we face epistemic uncertainty regarding the authenticity of an individual with unstable values. In cases of such uncertainty in which we cannot rely on the patient’s own values, Nyholm and O’Neill suggest that

we may instead need to take as our reference points widely endorsed values that are viewed as sensible or legitimate even by those who do not hold them: the commonly recognized range of what are regarded as values about which there can be reasonable discussions and disagreements. If the values the patient has in one mind-set fall squarely outside of this range, whereas the values the patient has in a different mind-set fall inside of this range, then this might be taken to give us reason to suppose that the latter values are more expressive of the person’s true self than are the former.^48^

We recognize the appeal of this strategy in that it provides us with a clear action-guiding principle in cases of epistemic uncertainty. However, the previous reflections suggest that more work needs to be done on explicating *why* the fact that a mind-set incorporating values that fall inside the range of widely endorsed values should be understood as the mind-set that is more expressive of the person’s true self. This sort of view seems inimical to Mill’s championing of individuality against the forces of custom, and his derisory claim that “he who lets the world, or his own portion of it, choose his plan of life for him, has no need of any other faculty than the ape-like one of imitation.”^49^ We do not deny that a case can be made in favor of Nyholm and O’Neill’s claim; our point here is that it seems prima facie problematic to ascertain authenticity, a concept whose value is tied to individual meaning, by reference to the *values of others*. This is not merely a pedantic theoretical foible. In light of the close relationship between authenticity and autonomy according to many approaches (including our own), the identification of authentic desires as those that are congruous with widely shared values raises the prospect that this strategy might in practice amount to dressing up considerations of beneficence in the language of autonomy; this in turn, is a good recipe for paternalism, albeit via the back door.

## Conclusion

We have defended a coherentist approach to authenticity that draws on both existentialist and essentialist themes. It grounds claims of authenticity by an appeal to the agent’s diachronic values, recognizing that such values, although not immutable, are likely to be long-lasting and difficult to change. We believe that this diachronic approach is better placed to respond to Erler and Hope’s critique of existentialist approaches to authenticity than the synchronic approach outlined by Nyholm and O’Neill. Although the approach that we have defended denies the presence of a hidden essential coherent self that requires discovery, the coherentist approach can offer practical guidance to those who wish to invoke the language of authenticity in their practical deliberations. When considering whether some element of the self is authentic, we must consider not just whether the individual rationally endorses it, but also whether that evaluation is incorporated into a coherent character system, whose lineage can be traced back over a diachronic process of intelligible rational change. We have also drawn attention to the conflicting nature of character traits, and how authenticity may involve greater emphasis on some, and downplaying others.

This account will not provide us with a “one- size-fits-all” answer to questions of authenticity in mental disorder, or to questions regarding the implications of DBS for authenticity; much will depend on how individual agents view their own condition in their self-conception and their other evaluations, and whether DBS is most aptly construed as effecting their traits or their values themselves. However, we take this flexibility to be a strength of our approach, in that it is able to adapt to the individual experiences of psychiatric disorders and treatment. If we are serious about protecting the value of individuality that seems to be at the heart of authenticity, then we believe that there is good reason to be wary of less flexible approaches, in so far as they threaten to impose an objective conception of the good onto others in the name of their authenticity.

## Notes

1. Mayberg HS, Lozano AM, Voon V, McNeely HE, Seminowicz D, Hamani C. Deep brain stimulation for treatment-resistant depression. *Neuron* 2003;45(5):651–60; Lipsman N, Lozano AM. Targeting emotion circuits with deep brain stimulation in refractory anorexia nervosa. *Neuropsychopharmacology* 2014;39(1):250–51; Bari AA, Kon Kam King N, Lipsman N, Lozano AM. Deep brain stimulation for neuropsychiatric disorders. In: Tuszynski MH, ed. *Translational Neuroscience*. New York: Springer US;2016:499–516.

2. Rodriguez-Oroz MC, Obeso JA, Lang AE, Houeto JL, Pollak P, Rehncrona S. Bilateral deep brain stimulation in Parkinson’s disease: A multicentre study with 4 years follow-up. *Brain* 2005;128(10): 2240–49; Hu W, Stead M, Deep brain stimulation for dystonia. *Translational Neurodegeneration* 2014;3:2.

3. Clausen J. Ethical brain stimulation – neuroethics of deep brain stimulation in research and clinical practice. *European Journal of Neuroscience* 2010;32(7):1152–62.

4. BaylisF. ’I am who I am’: On the perceived threats to personal identity from deep brain stimulation. Neuroethics 2013;6(3):513–262427362110.1007/s12152-011-9137-1PMC3825414; Kraemer F. Me, myself and my brain implant: Deep brain stimulation raises questions of personal authenticity and alienation. *Neuroethics* 2013;6(3):483–97; Lipsman N, Glannon W. Brain, mind and machine: What are the implications of deep brain stimulation for perceptions of personal identity, agency and free will? *Bioethics* 2013;27(9):465–70; Klaming L, Haselager P. Did my brain implant make me do it? Questions raised by DBS regarding psychological continuity, responsibility for action and mental competence, *Neuroethics* 2010;6(3):527–39.

5. See note 4, Kraemer 2013; Nyholm S, O’Neill E. Deep brain stimulation, continuity over time, and the true self. Cambridge Quarterly of Healthcare Ethics 2016;25(4):647–5810.1017/S096318011600037227634716; MaslenH, PughJ, SavulescuJ. Authenticity and the stimulated self: Neurosurgery for anorexia nervosa. AJOB Neuroscience 2015;6(4):69–71.

6. See note 4, Baylis 2013.

7. SharpD, WassermanD. Deep brain stimulation, historicism, and moral responsibility. Neuroethics 2016;9(2):173–85; see note 4, Klaming, Haselager 2010.

8. See note 4, Kraemer 2013.

9. The issues pertaining to personal identity and moral responsibility might also be understood to be more complicated in the case of psychiatric disorders to the extent that such disorders can be understood to threaten personal identity and/or moral responsibility. However, we do not make a stand on this claim here.

10. See note 4, Kraemer 2013.

11. MaslenH, PughJ, SavulescuJ. The ethics of deep brain stimulation for the treatment of anorexia nervosa. Neuroethics 2015;8(3):215–2302659425610.1007/s12152-015-9240-9PMC4643100; see note 15, Maslen et al. 2015.

12. See note 5, Nyholm, O’Neill 2015.

13. Marya Schetmann calls this the characterization question. See Schechtman M. *The Constitution of Selves*. Ithaca; London: Cornell University Press; 1996, 73.

14. We note that the spatial metaphor of peripheral traits is suggestive of synchronicity: traits at different distances from the central self, instantiated at the same time. However, we do not intend to use this metaphor to illustrate anything substantive about the account of authenticity we will develop: peripheral traits might be better or alternatively understood as less frequently instantiated.

15. Newman GE, Bloom P, Knobe J. Value judgments and the true self. *Personality and Social Psychology* 2014;40(2):203–16.

16. See note 15, Strohminger et al.

17. See note 15, Strohminger et al.; DeGrazia D. *Human Identity and Bioethics*. Cambridge: Cambridge University Press; 2005, at 233–34.

18. In their discussion, Erler and Hope draw a tripartite distinction between what they term “authenticity as wholeheartedness,” “authenticity as autonomous and honest endorsement,” and “true-self-accounts.” The first two can be understood to be examples of what we call existentialist accounts, whereas the latter maps onto what we are terming essentialist accounts. Erler A, Hope T. Mental disorder and the concept of authenticity. *Philosophy, Psychiatry, & Psychology* 2015;21(3):219–32.

19. BublitzJC, MerkelR. Autonomy and authenticity of enhanced personality traits. Bioethics 2009;23(6):370.10.1111/j.1467-8519.2009.01725.x19527264

20. See note 18, Erler, Hope 2015.

21. Parens E, Authenticity and ambivalence: Toward understanding the enhancement debate. *The Hastings Center Report* 2005;35(3):34–41.

22. Levy N. Enhancing authenticity. *Journal of Applied Philosophy* 2011;28(3):312.

23. See note 5, Nyholm, O’Neill, 2016.

24. Elliott C. *Better than Well : American Medicine Meets the American Dream*. New York ,London: W.W. Norton; 2003.

25. Levy N. Enhancing authenticity. *Journal of Applied Philosophy* 2011;28(3):316.

26. Schechtman M. *The Constitution of Selves*. Ithaca; London: Cornell University Press; 1996.

27. See note 17, DeGrazia 2005, at 102.

28. See note 18, Erler, Hope 2015.

29. For a detailed account of this rationalist approach see See Parfit D. *On What Matters*. Oxford: Oxford University Press; 2011. Part One.

30. EkstromLW. A coherence theory of autonomy. Philosophy and Phenomenological Research 1993;53(3):599–616.

31. See note 30, Ekstrom 1993, at 607.

32. See note 30, Ekstrom 1993, at 608–9.

33. According to social psychology research, we may also observe that people would likely attribute authenticity to Scrooge by virtue of the fact that they regard his change in character as having positive valence. See note 15, Strohminger et al.

34. Robert Noggle also appeals to the idea of “Neurathian Autonomy” in Noggle R. The public conception of autonomy and critical self-reflection. *Southern Journal of Philosophy* 1997;35(4):510. However, he appeals to this sort of idea with regards to what he terms the core attitudes that undergird agential autonomy. See Noggle R. Autonomy and the paradox of self-creation. In: Taylor JS, ed. *Personal Autonomy New Essays on Personal Autonomy and Its Role in Contemporary Moral Philosophy*, Cambridge: Cambridge University Press; 2005.

35. See note 30, Ekstrom 1993, at 600.

36. DoshiP, BhargavaP. Hypersexuality following subthalamic nucleus stimulation for Parkinson’s disease. Neurology India 2008;56(4):474–76.1912704510.4103/0028-3886.44830

37. Voon V, Kubu C, Krack P, Houeto JL, Tröster AI. Deep brain stimulation: Neuropsychological and neuropsychiatric issues. *Movement Disorders* 2006;21(Suppl 14):S305–327.

38. Tan J, Hope T, Stewart A, Fitzpatrick R. Competence to make treatment decisions in anorexia nervosa: Thinking processes and values. *Philosophy, Psychiatry, & Psychology* 2007;13(4):267–82.

39. See note 11, Maslen et al. 2015.

40. FocquaertF, SchermerM. Moral enhancement: Do means matter morally? Neuroethics 2015;8(2): 139–51.10.1007/s12152-017-9340-9PMC556913528890740

41. See note 11, Maslen et al. 2015.

42. See note 18, Erler, Hope 2015.

43. See note 4, Kraemer 2013.

44. Mill JS. *On Liberty*. New Haven: Yale University Press; 2003, at 125.

45. See note 15, Strohminger et al.

46. See note 41, Mill 2003, at 131.

47. Hughes J. Beyond “Real Boys” and Back to Parental Obligations. *The American Journal of Bioethics* 2005;5(3):61.

48. See note 5, Nyholm, O’Neill 2015.

49. See note 41, Mill 2003, at 124.

